# CXCL1 Regulated by miR-302e Is Involved in Cell Viability and Motility of Colorectal Cancer *via* Inhibiting JAK-STAT Signaling Pathway

**DOI:** 10.3389/fonc.2020.577229

**Published:** 2021-05-17

**Authors:** Biyin Chen, Li Song, Xiuzhen Nie, Fangfeng Lin, Zongyang Yu, Wencui Kong, Xiaoyan Qi, Wenwu Wang

**Affiliations:** ^1^ Department of Oncology, The Third Affiliated People’s Hospital of Fujian University of Traditional Chinese Medicine, Fuzhou, China; ^2^ Department of Pulmonary and Critical Care Medicine, The 900th Hospital of Joint Logistic Support Force, PLA, Fuzhou, China; ^3^ Department of Oncology, Zibo Central Hospital, Zibo, China

**Keywords:** miR-302e, chemokine ligand-1, janus kinase signal transducer and activator of transcription, colorectal cancer, proliferation, migration and invasion

## Abstract

**Purpose:**

This study made a systemic description for the CXCL1-dependent regulatory mechanism in colorectal cancer (CRC).

**Methods:**

Bioinformatics methods were applied to obtain target mRNA CXCL1 and corresponding upstream miRNA. qRT-PCR and Western blot were performed to measure the levels of CXCL1 and miR-302e in CRC tissue and cells. Experiments including CCK-8, wound healing assay, Transwell invasion assay, and flow cytometry were conducted to assess cell biological behaviors. Dual-luciferase reporter assay was carried out for verification of the targeting relationship between CXCL1 and miR-302e. The inhibitor AG490 of JAK-STAT signaling pathway was used to identify the functional mechanism of CXCL1/JAK-STAT underlying progression of CRC, and tumor xenograft experiments were performed for further validation.

**Results:**

CXCL1 was highly expressed in CRC tissue and cells, while miR-302e was poorly expressed. Silencing CXCL1 or overexpressing miR-302e could lead to inhibition of cell proliferation, migration, invasion but promotion of cell apoptosis of CRC. Besides, CXCL1 was identified as a direct target of miR-302e, and CXCL1 could reverse the effect of miR-302e on cell proliferation, migration, invasion, and apoptosis. Furthermore, CXCL1 functioned on CRC cell biological behaviors *via* activation of JAK-STAT signaling pathway.

**Conclusion:**

CXCL1 could be regulated by miR-302e to inactivate JAK-STAT signaling pathway, in turn affecting cell proliferation, migration, invasion, and apoptosis of CRC. Our result provides a potential therapeutic target for CRC treatment.

## Introduction

Colorectal cancer (CRC) is the third most common cancer worldwide and the fourth leading cause resulting in cancer death (1). In 2018, approximately 1,800,000 people were diagnosed with CRC and the deaths were up to 881,000 (2), which posed a threat to human life and health. Currently, the predominant curative option for CRC is surgery with chemotherapy in the second place, yet the 5-year overall survival remains very poor (3, 4). The main cause can be attributed to that liver as well as lung metastasis is much easier to develop in advanced CRC (5), and that is the major reason for patients failing to receive operative treatment (6). Hence, it prompts us to know more about the pathogenesis and underlying molecular mechanisms of CRC tumor metastasis, which will help find potential therapeutic targets for CRC treatment.

MicroRNAs (miRNAs) are endogenous non-coding RNA molecules with a length about 22 nucleotides, and they are involved in messenger RNA (mRNA) degradation or translational inhibition by means of interacting with the 3′-untranslated regions (3′-UTR) within their target mRNAs (7–9). Prior studies revealed that miRNAs are important players involved in occurrence and progression of CRC, including cell proliferation, tumor metastasis, differentiation of cancer stem cells and cell apoptosis. miR-215-3p, for example, can suppress the growth, migration and invasion of CRC (10), while miR-452 can promote cell proliferation, migration, and angiogenesis (11), and another miRNA, miR-376a-3p, is beneficial for CRC progression (12). In addition, Li D *et al*. reported that long non-coding RNA FGD5-AS1 could sponge miR-302e to up-regulate the level of CDCA7, in turn potentiating the proliferation and migration of CRC cells. In the present study, we also found that miR-302e was able to make an effect on cell biological properties, which further identifies its role as a potential therapeutic target for CRC treatment.

Chemokine ligand-1 (CXCL1), a member of CXC chemokine family, was first detected in melanoma and can be expressed in macrophages, neutrophils and epithelial cells. CXCL1 can exert its role in neutrophil chemotaxis *via* specifically interacting with its receptor CXCR2, meanwhile, it is involved in some processes, such as spinal development, inflammation, angiogenesis, and wound healing (13–15). As research goes on, CXCL1 has been identified to be intimately associated with the development of various cancers. For instance, Chengcheng Yang et al. (16) found that CXCL1 could induce activation of ERK/MMP2/9 signaling axis to stimulate cell migration and invasion of ER-negative breast cancer. Lu Y et al. (17) indicated that CXCL1-LCN2 axis is able to positively function on the development of prostate cancer *via* boosting Src signaling pathway activation as well as epithelial-mesenchymal transition process. In addition, the role of CXCL1 in CRC has also been researched. For example, CXCL1 potentiated CRC metastasis by interacting with CXCR2, as suggested by Dingzhi Wang et al. (18). Nevertheless, specific mechanisms of CXCL1 have not been fully studied in CRC.

In the present study, we validated that CXCL1 was differentially expressed in CRC and it was involved in cell proliferation, metastasis, and apoptosis. Meanwhile, we further verified that there was a targeting relationship between CXCL1 and miR-302e, and CXCL1 could positively function on CRC occurrence and progression *via* inducing activation of JAK-STAT signaling pathway. Our results can help explore a novel strategy for CRC treatment.

## Materials and Methods

### Bioinformatics Analysis

GEO database (https://www.ncbi.nlm.nih.gov/geo/) was used for acquisition of CRC microarrays, including GSE41328 (normal: n = 10; tumor: n = 10), GSE75970 (normal: n = 4; tumor: n = 4), and GSE89076 (normal: n = 39; tumor: n = 41). Following batch adjustment using the “sva” package in R, the obtained expression data were subjected to differential analysis using the “limma” package to identify differentially expressed genes (DEGs) that met |logFC|>2 and FDR<0.05 (*p* value was adjusted based on FDR method). Afterwards, STRING database (https://string-db.org/) was used to construct a protein-protein interaction (PPI) network which was then visualized on Cytoscape 3.7.1. UALCAN database was applied to analyze CXCL1 expression in TCGA-COAD (colon adenocarcinoma) and TCGA-READ (rectum adenocarcinoma) datasets. TargetScan (http://www.targetscan.org/vert_71/), mirDIP (http://ophid.utoronto.ca/mirDIP/index.jsp#r), miRDB (http://mirdb.org/), miRSearch (https://www.exiqon.com/miRSearch), and microRNA (http://www.microrna.org/microrna/home.do) databases were used to predict upstream miRNAs for CXCL1, while the microRNA database was consulted to identify the binding sites of the target miRNA on CXCL1 3′UTR fragments.

### Sample Collection

A panel of clinical CRC tissue samples (n = 56) and matched adjacent normal tissue samples (n = 56, >2 cm in margin) were obtained from patients who were pathologically diagnosed with CRC and underwent surgery treatment from October 2015 to October 2019 in the Third Affiliated Hospital of Fujian University of Traditional Chinese Medicine. Corresponding clinical information of all the samples were available, and all the subjects received no preoperative chemotherapy or radiotherapy. This study had gained the informed consent from each subject and been approved by the Ethic Committee of the Third Affiliated Hospital of Fujian University of Traditional Chinese Medicine.

### Cell Culture

Human normal colorectal mucosa cell line FHC (ATCCCRL-1831) and human CRC epithelial cell lines SW837 (ATCCCCL-235), SW480 (ATCCCCL-228), Caco2 (BNCC350772), and HT29 (ATCCHTB-38) were all ordered from American Type Culture Collection (ATCC, Manassas, VA). Dulbecco’s Modified Eagle Medium (DMEM; Gibco, Grand Island, NY, USA) supplemented with 10% fetal bovine serum (FBS; Gibco, Grand Island, NY, USA) was used for cell culture at 37°C and in 5% CO_2_.

### Cell Transfection

The miR-302e-mimic, miR-302e-inhibitor, oe-CXCL1, short hairpin RNA (shRNA) targeting CXCL1 (sh-CXCL1) and their negative controls (NC) purchased from GeneChem (Shanghai, China) were transfected into CRC cells using Lipofectamine^®^ 2000 reagent (Invitrogen, Carlsbad, USA) per the manufacturer’s instructions. The oe-CXCL1 designed by GeneChem (Shanghai, China) was cloned into GV112 lentivirus vector (GeneChem, Shanghai, China) for preparation and then used to infect cells with polybrene. 10 μM of AG490 (an inhibitor against JAK-STAT signaling pathway) (Selleck Chemicals, Houston, TX, U.S.A.) or vehicle (DMSO) was transected into cells as indicated. After 48 h, the cells were exposed to 1 mg/ml of puromycin for screening stable colonies.

### Real-Time Quantitative PCR

Total RNA was extracted from frozen tissues and cells using Trizol reagent (Invitrogen, Carlsbad, USA). Thereafter, the RNA was treated with the One Step miRNA cDNA Synthesis Kit (Invitrogen, Carlsbad, USA) for miRNA cDNA synthesis and with the Thermo Scientific RevertAid First Strand cDNA Synthesis Kit (Invitrogen, Carlsbad, USA) for mRNA cDNA synthesis. qRT-PCR was run on the Applied Biosystems 7500 instrument (Applied Biosystem) with the SYBR Premix Ex Taq Assay Kit (TaKaRa, Dalian, China). Expression levels of miR-302e, miR-520a-3p, miR-520c-3p, and miR-520d-3p were normalized to U6 *via* 2^-ΔΔCt^ method, and level of CXCL1 mRNA was normalized to GAPDH. See **Table 1** for detailed PCR primer sequences.

**Table 1:** Primer sequence.

ID	Primer sequence
miR-302e	Forward : 5′-CTCATCGCATAAGTGCTTCCAT-3′
	Reverse : 5′-TATCGTTGTTCTCGACTCCTTCAC -3′
miR-520a-3p	Forward : 5′-ACACTCCAGCTGGGAAAGTGCTTCCC-3′
	Reverse : 5′-CTCAACTGGTGTCGTGGA-3′
miR-520c-3p	Forward : 5′-GCCGCCAAAGTGCTTCCTTTTAG-3′
	Reverse : 5′-TCGCACTGGATACGACACCCTC-3′
miR-520d-3p	Forward : 5′-GGTCTACAAAGGGAAGC-3′
	Reverse : 5′-TTTGGCACTAGCACATT-3′
U6	Forward : 5′-CTCGCTTCGGCAGCACATATACTA-3′
	Reverse : 5′-ACGAATTTGCGTGTCATCCTTGCG-3′
CXCL1	Forward : 5′- CTCGAGGCCCCTGGGGCAGAAGCCTC -3′
	Reverse : 5′- GATATCGGGGCTCAGCAGGCGGGTCT -3′
GADPH	Forward : 5′-TATGATGATATCAAGAGGGT AGT-3′
	Reverse : 5′-TGTATCCAAACTCATTGTCATAC-3′

### Western Blot

After 48 h of transfection, the cells were washed with pre-cooled PBS (Thermo Fisher Scientific, MA, USA) 3 times and then lysed in RIPA lysis buffer (Thermo Fisher Scientific, MA, USA) on ice for 10 min. The BCA assay kit from Thermo Fisher Scientific (MA, USA) was applied for quantification of the protein samples. After boiled at 95°C for 10 min, the protein samples were subjected to sodium dodecyl sulfate polyacrylamide gel electrophoresis (SDS-PAGE) at 100V and then transferred to nitrocellulose membranes. Five percent BSA/TBST was used to block the membranes for 60 min. Subsequently, the membranes were incubated with primary rabbit polyclonal antibodies overnight at 4°C and with horseradish peroxidase (HRP) -conjugated secondary antibody goat anti-rabbit (ab6721, 1:3000, abcam, Cambridge, UK) in succession for 120 min of hybridization at room temperature. 1 × TBST (Solarbio, Beijing, China) was used to wash the membranes. Protein bands were visualized using the electrochemiluminescence kit (Solarbio, Beijing, China) and photographed. Primary antibodies include CXCL1 (PAI-29220, 1:2000, Thermo Fisher Scientific), Jak2 (ab108596, 1:5000, abcam), p-Jak2 (ab76293, 1:5000, abcam), STAT3 (9133S, 1:1000, Cell Signaling Technology), p-STAT3 (8204S, 1:1000, Cell Signaling Technology), and GAPDH (ab181602, 1:10000, abcam).

### CCK-8

A measure of 200 μl of cell suspension (1 × 10^4^ cells/ml) was seeded into 96-well plates and five parallel wells were set for each treatment. After 0, 1, 2, and 3 days, 10 μl of CCK-8 was added into each well for cell incubation at a 37°C incubator for 2 h. Optical density (OD) values at 450 nm were measured with an enzyme labeled instrument (Multiskan MK3, Thermo) for determination of cell viability.

### Wound Healing Assay

Cells (2 ml, 3 × 10^5^ cells/ml) planted in 6-well plates were grown to 90% in confluence. Then, the cells were wounded by the tip of a sterile pipette. PBS was used for removing the separated cells and the cells remaining were continuously cultured in FBS-free mediums at 37°C in 5% CO_2_. The wounded areas at 0 and 48 h time points were observed under an inverted microscope and sequentially photographed. The wound healing rate was calculated using the Image J software.

### Transwell Invasion Assay

Transwell inserts (sigma, China) were firstly coated with Matrigel matrix (BD, USA) 4 h prior to invasion assay, and then filled with 200 μl of cell suspension (1×10^5^ cells/ml) that had been suspended by FBS-free mediums. Afterwards, the inserts were put into a 24-well plate that contained 10% FBS-supplemented mediums. After incubation in a 37°C incubator in 5% CO_2_ for 1 day, cells still in the inserts were softly wiped off using a cotton swab, whereas cells invading into the plate were harvested for 30 min of fixation with 4% polyformaldehyde followed by 30 min of staining in 0.1% crystal violet. Images were captured under an inverted microscope from five randomized fields and then the cell number was calculated.

### Flow Cytometry

For evaluation of cell apoptosis, 2 ml of cell suspension with a concentration of 1 × 10^5^ cells/ml was firstly inoculated into 6-well plates. After 72 h, the cells were harvested following 3 min of centrifugation at 1000 rpm, and sequentially washed with the buffer supplied by the BD apoptosis assay kit (BD Biosciences, USA), according to the standard process. FITC-Annexin V and PI were used for cell staining and flow cytometry (BD Biosciences, USA) was applied for cell apoptosis analysis. The Cell QuestPro software (BD Biosciences, USA) was employed for data analysis to calculate cell apoptotic rate.

### Dual-Luciferase Reporter Assay

Amplified sequences on the wild type or mutant 3′UTR of CXCL1 (3′UTR-Wt or 3′UTR-Mut) were cloned into pmirGLO luciferase vectors (Promega, WI, USA) to construct CXCL1-Wt or CXCL1-Mut plasmid vectors which were then validated by DNA sequencing. CXCL1-Wt and CXCL1-Mut plasmid vectors (pGL3 as control) were in succession co-transfected with miR-302e mimic or NC mimic into SW480 cells using Lipofectamine^®^ 2000, respectively. Forty-eight hours after transfection, the cells were harvested and the Luciferase activity (Renilla luciferase activity as control) was assayed using the Dual-Luciferase Reporter Assay System (Promega, WI, USA).

### Tumor Xenograft Assay

Thirty female BALB/c nude mice (4–6-week-old, 15–20 g) were provided by Shanghai Institute of Materia Medica, Chinese Academy of Sciences (Shanghai, China) with the production license as SCXK (Shanghai) 2018-0002. All the mice were bred under sterile conditions and housed in a certain environment (12 h/12 h light/dark cycle, 25°C, 60%–70% humidity). The nude mice were divided into three groups with 10 mice per group for further observation. For *in vivo* proliferation assay, an estimate of 1 × 10^7^ SW480 cells of different treatments were subcutaneously injected into the nude mice of three groups. Tumor volume (V) was monitored every week and calculated by measuring the length (L) and width (W) with the formula as V (mm^3^) = L × W^2^/2. After 4 weeks, all the mice were euthanatized and the xenograft tumors were isolated for weighing and photographs capture.

### Immunohistochemistry

Paraffin-embedded xenograft tumor tissue sections were pre-treated in boiled water with sodium citrate buffer (pH6.0) for 30 min and then blocked in 1% BSA at room temperature for 1 h. Thereafter, the sections were incubated with the following primary antibodies at 4°C overnight: CXCL1 (PA5-86508, 1:200, Invitrogen) and Ki67 (ab833, 1:50, abcam), followed by secondary antibody goat anti-rabbit IgG (1:200, A-21239, Invitrogen) at room temperature for 2 h. Finally, the sections were stained with eosin and hematoxylin, and then washed, dehydrated and sealed for further observation. Primary antibodies were not used in the negative control group.

### Statistical Analysis

All data obtained in this study were processed on the GraphPad Prism 7 software (GraphPad Software, Inc., La Jolla, CA). The form of mean ± standard deviation was used for presentation of measurement data. Student’s *t* test and one-way analysis of variance (ANOVA) were applied for analyzing the comparisons between two groups or among multiple groups, while ANOVA for repeated measurement data was employed for the comparisons among different time points, with the Turkey’s test used for further verification. All experiments were repeated at least three times. *P*<0.05 was considered statistically significant.

## Results

### Analysis of DEGs in CRC Using Bioinformatics Methods

**Figure 1:**
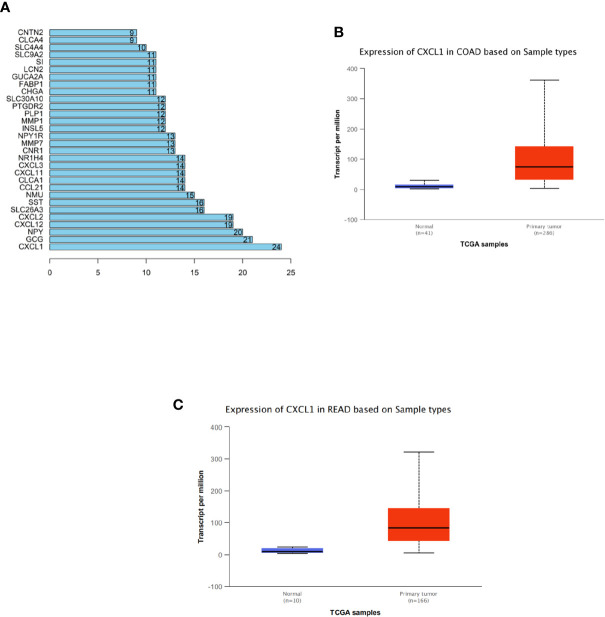
Analysis of DEGs in CRC *via* bioinformatics methods. **(A)** Degrees of the top 30 DEGs among all the screened CRC-related differential genes from GEO. The abscissa refers to degree and the ordinate refers to the gene, **(B, C)** Expression levels of CXCL1 in TCGA-COAD (colon adenocarcinoma) and TCGA-READ (rectum adenocarcinoma) datasets (blue: normal; red: tumor). The abscissa refers to sample type and the ordinate refers to the transcript per million.

Totally, 294 CRC-related DEGs were obtained *via* differential analysis (**Supplementary Figure 1**). Then, these DEGs were used to construct a PPI network (**Supplementary Figure 2**). The degree (number of interacted genes) of each gene was calculated (**Supplementary Material 1**) and eventually 30 DEGs with a relatively high degree were identified as shown in **Figure 1A**. Among the 30 DEGs, CXCL1 was identified as the hub gene for the highest degree. In addition, further analysis of CXCL1 in samples from TCGA-COAD and TCGA-READ datasets revealed that CXCL1 expression in tumor tissue was significantly elevated relative to that in normal tissue (**Figures 1B, C** **)**. Hence, we initially concluded that CXCL1 was highly expressed in CRC and high expression of CXCL1 might promote CRC occurrence.

### CXCL1 Is Highly Expressed in CRC Tissue and Cells

**Figure 2:**
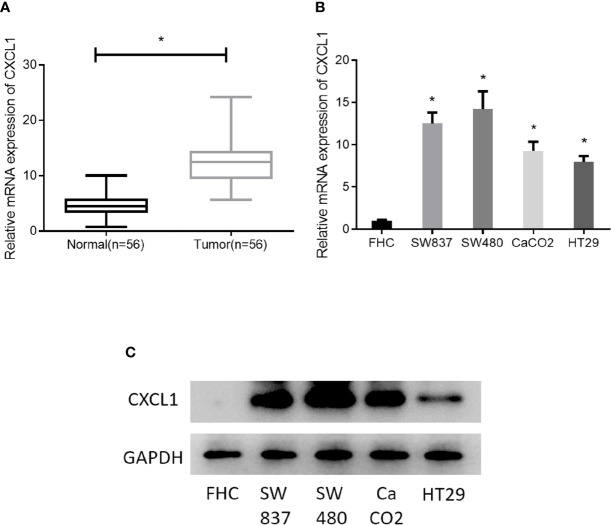
CXCL1 is highly expressed in CRC. **(A)** qRT-PCR results showed the expression of CXCL1 in clinical tissue samples (normal: n = 56; tumor: n = 56), **(B, C)** Expression of CXCL1 mRNA and proteins in normal colorectal mucosa cell line FHC and CRC cell lines SW837, SW480, CaCO2, HT29, as examined by qRT-PCR and Western blot (*p < 0.05).

As revealed by bioinformatics analysis, we had initially known that CXCL1 was highly expressed in CRC. For further verification, 56 pairs of clinical CRC tissue samples and adjacent normal tissue samples were collected for CXCL1 expression detection, finding that the expression of CXCL1 in tumor tissue was significantly higher than that in adjacent tissue (**Figure 2A**). In addition, CXCL1 was also assessed in four CRC cell lines SW837, SW480, CaCO2, HT29, and one normal colorectal mucosa cell line FHC. Similarly, CXCL1 was remarkably increased in both mRNA and protein levels in cancer cell lines relative to that in the normal cell line (**Figures 2B, C** **)**. In view of these, the result obtained by bioinformatics analysis was credible, and it could be reasoned that high expression of CXCL1 might pose a positive effect on CRC development.

### Silencing CXCL1 Inhibits Cell Proliferation, Migration, Invasion, and Promotes Cell Apoptosis of CRC

**Figure 3:**
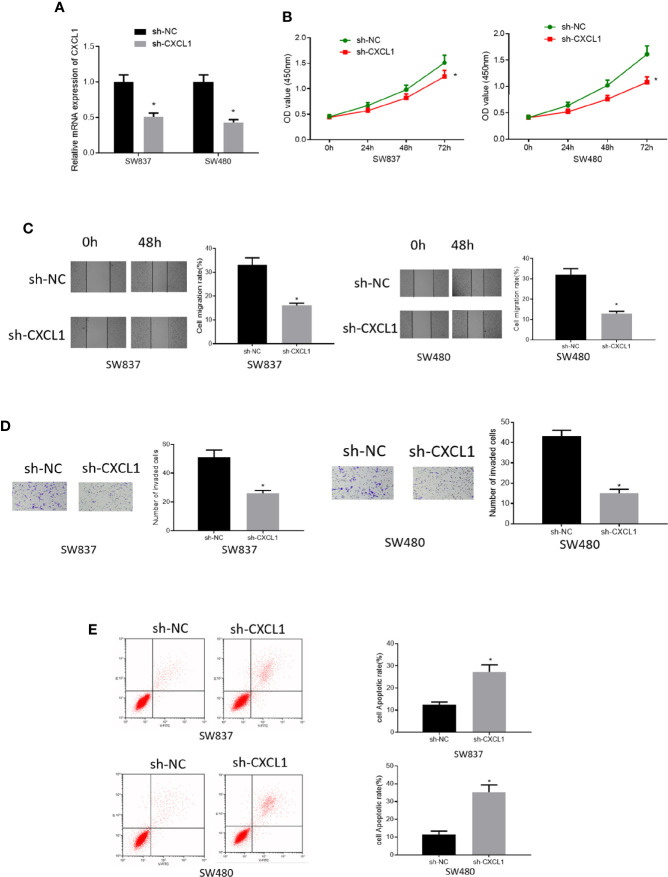
Silencing CXCL1 inhibits cell proliferation, migration, invasion and promotes cell apoptosis of CRC. **(A)** qRT-PCR results showed the relative expression of CXCL1 after sh-CXCL1 and sh-NC were respectively transfected into SW837 and SW480 cells, **(B)** CCK-8, **(C)** wound healing assay, **(D)** Transwell invasion assay (100×), and **(E)** flow cytometry results showed the effect of silenced CXCL1 on proliferation, migration, invasion, and apoptosis of transfected cells (*p < 0.05).

To investigate the role of CXCL1 in CRC, SW837, and SW480 cell lines with relatively high expression of CXCL1 were selected for follow-up analysis. Sh-CXCL1 was transfected into the two cell lines, respectively, to realize CXCL1 knockdown. qRT-PCR was performed to determine the interference efficiency and it was showed that CXCL1 was successfully decreased in sh-CXCL1 transfected cells (**Figure 3A**). Then, cell biological behaviors were assayed *via* some *in vitro* experiments. CCK-8 results suggested that down-regulation of CXCL1 resulted in decrease of cell viability, and similar effect was observed on cell migratory and invasive abilities as detected by wound healing assay and Transwell invasion assay (**Figures 3B–D**). Meanwhile, cell apoptosis was tested by flow cytometry and the results plotted in **Figure 3E** presented that cell apoptotic rate in sh-CXCL1 treated cells was greatly increased. Taken together, these findings demonstrated that CXCL1 acted as an oncogene in CRC to exert its role in regulating cell proliferation, migration, invasion and apoptosis.

### miR-302e Inhibits CXCL1 Expression in CRC Cells

**Figure 4:**
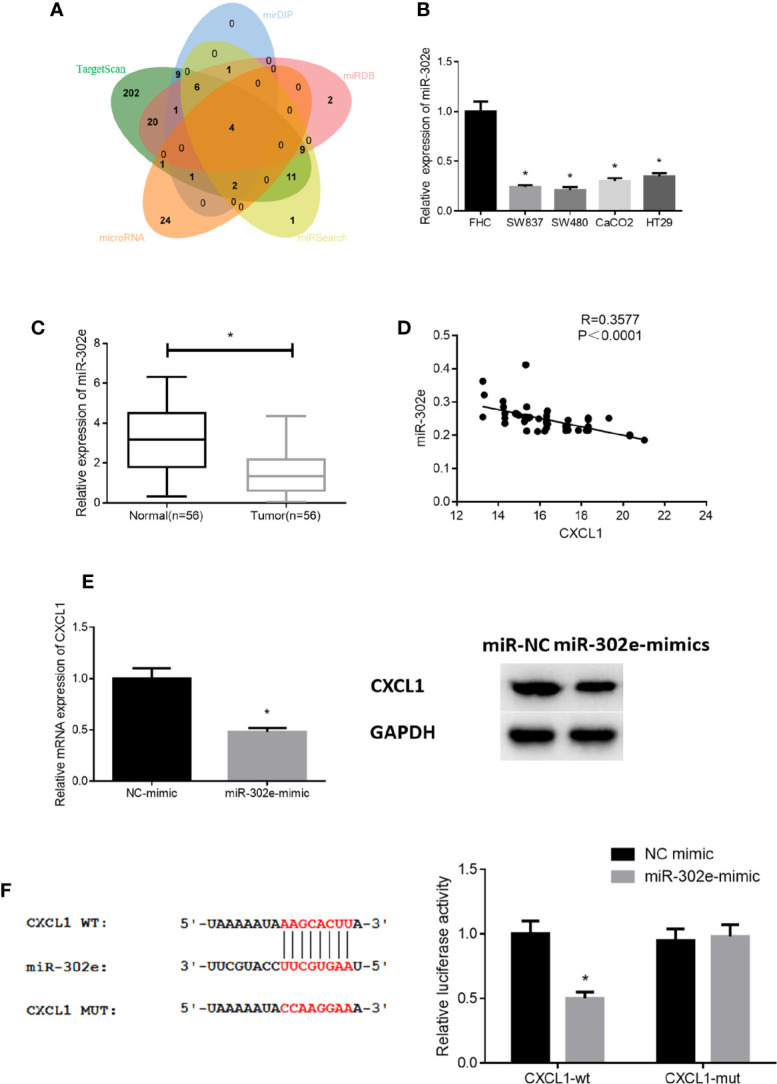
miR-302e targets CXCL1 in CRC cells. **(A)** Venn diagram shows the overlapping miRNAs from genes predicted by TargetScan, mirDIP, miRDB, miRSearch, and microRNA databases, **(B, C)** qRT-PCR results showed the expression level of miR-302e in **(B)** CRC cell lines SW837, SW480, CaCO2, HT29, and normal colorectal mucosa cell line FHC, as well as in **(C)** clinical tumor tissue samples and adjacent normal tissue samples (n = 56), **(D)** Pearson correlation analysis showed that there was a negative correlation between CXCL1 and miR-302e levels, **(E)** qRT-PCR and Western blot were conducted to show the relative mRNA and protein expression of CXCL1 after miR-302e mimic and mimic NC were transfected into SW480 cells, **(F)** Dual-luciferase reporter assay was performed to validate the direct targeting relationship between miR-302e and CXCL1 (*p < 0.05).

To deeply explore the specific mechanism of CXCL1 in CRC, we applied bioinformatics analysis and found 4 miRNAs (miR-302e, miR-520a-3p, miR-520c-3p, miR-520d-3p) that might target CXCL1 (**Figure 4A**). qRT-PCR results revealed that miR-302e, miR-520a-3p and miR-520c-3p levels were all significantly decreased in CRC cases relative to those in normal cells, whereas miR-520d-3p expression was not obviously altered (**Figure 4B**). Due to the most significant alteration, miR-302e was chosen for further experiments. We then validated the expression of miR-302e in clinical CRC tissue samples and noted that it was greatly down-regulated in cancer tissue (**Figure 4C**). In addition, Pearson correlation analysis was conducted and it was found that there was a negative correlation between the levels of CXCL1 and miR-302e (**Figure 4D**), which is in accordance with the established miRNA-mRNA regulatory axis. Subsequently, miR-302e was overexpressed in SW480 cells (with the highest CXCL1 expression), finding that miR-302e overexpression prominently decreased the expression of CXCL1 mRNA and proteins, which indicated that CXCL1 might be negatively modulated by miR-302e in CRC cells (**Figure 4E**). Furthermore, dual-luciferase reporter assay was performed to confirm whether CXCL1 is a direct target of miR-302e, and it was noted that miR-302e inhibitory functioned on expression of CXCL1-Wt, but exhibited no effect on CXCL1-Mut (**Figure 4F**). Collectively, it was fully indicated that miR-302e could target and inhibit the expression of CXCL1 in CRC cells.

### miR-302e Suppresses Cell Proliferation, Migration, Invasion, and Potentiates Cell Apoptosis of CRC *via* Targeting CXCL1

**Figure 5:**
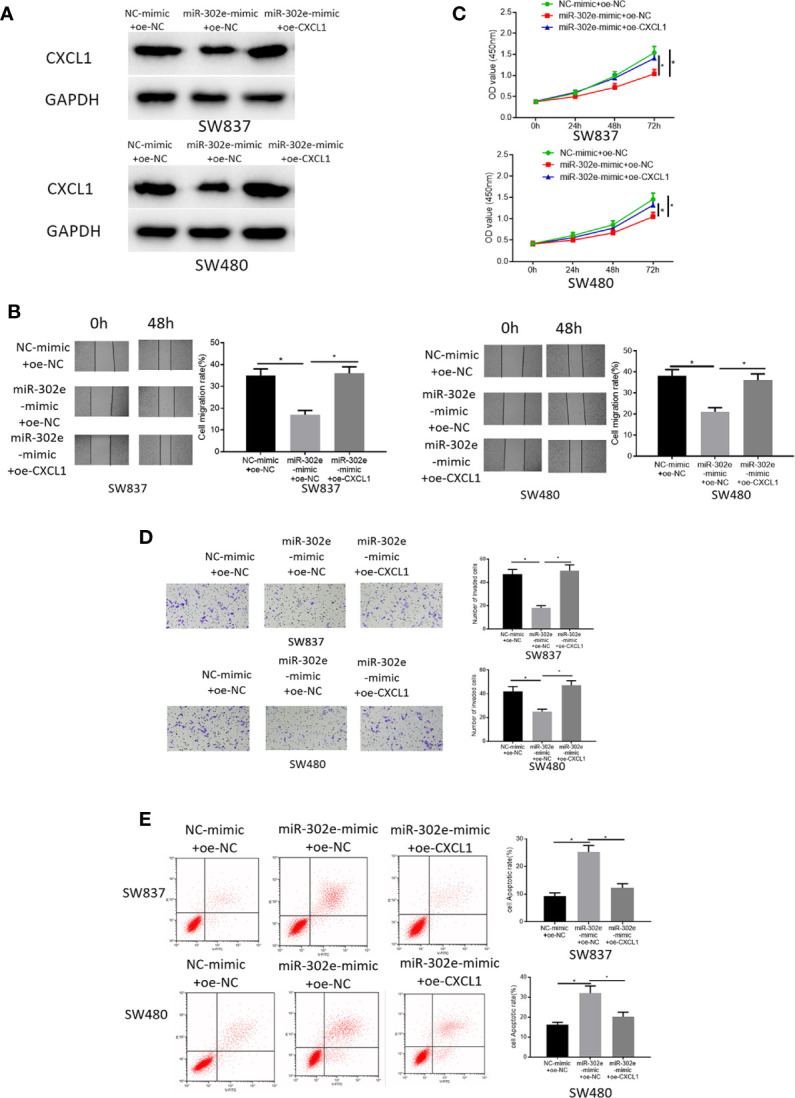
miR-302e targets CXCL1 to inhibit cell proliferation, migration, invasion and promote cell apoptosis of CRC. Cells were classified into NC-mimic+oe-NC, miR-302e-mimic+oe-NC, miR-302e-mimic+oe-CXCL1 groups. **(A)** Western blot experiment showed the protein expression of CXCL1 in SW873 and SW480 cells in each treatment group, **(B)** CCK-8, **(C)** wound healing assay, **(D)** Transwell invasion assay (100×), and **(E)** flow cytometry showed the proliferation, migration, invasion and apoptosis of SW873 and SW480 cells in each group (*p < 0.05).

As abovementioned, CXCL1 was a direct target of miR-302e. Hence, to clarify the functional mechanism of miR-302e/CXCL1 in CRC, NC-mimic+oe-NC, miR-302e-mimic+oe-NC, and miR-302e-mimic+oe-CXCL1 were respectively transfected into SW873 and SW480 cells. The results of Western blot experiments showed that overexpression of miR-302e inhibited the expression of CXCL1 in CRC cells, while with the presence of oe-CXCL1, CXCL1 expression was increased (**Figure 5A**). Additionally, as detected by CCK-8, wound healing assay and Transwell invasion assay, miR-302e overexpression inhibitory functioned on cell proliferation, migration and invasion, and such inhibition was rescued by overexpressed CXCL1 (**Figures 5B–D**). Reversely, cell apoptosis was enhanced when miR-302e was overexpressed but reversed as well by overexpressing CXCL1 (**Figure 5E**). Overall, we could see that miR-302e targeted CXCL1 to inhibit cell proliferation, migration, invasion, and promote cell apoptosis of CRC.

### CXCL1 Promotes Cell Proliferation, Migration, Invasion, and Inhibits Cell Apoptosis of CRC *via* Activating JAK-STAT Signaling Pathway

**Figure 6:**
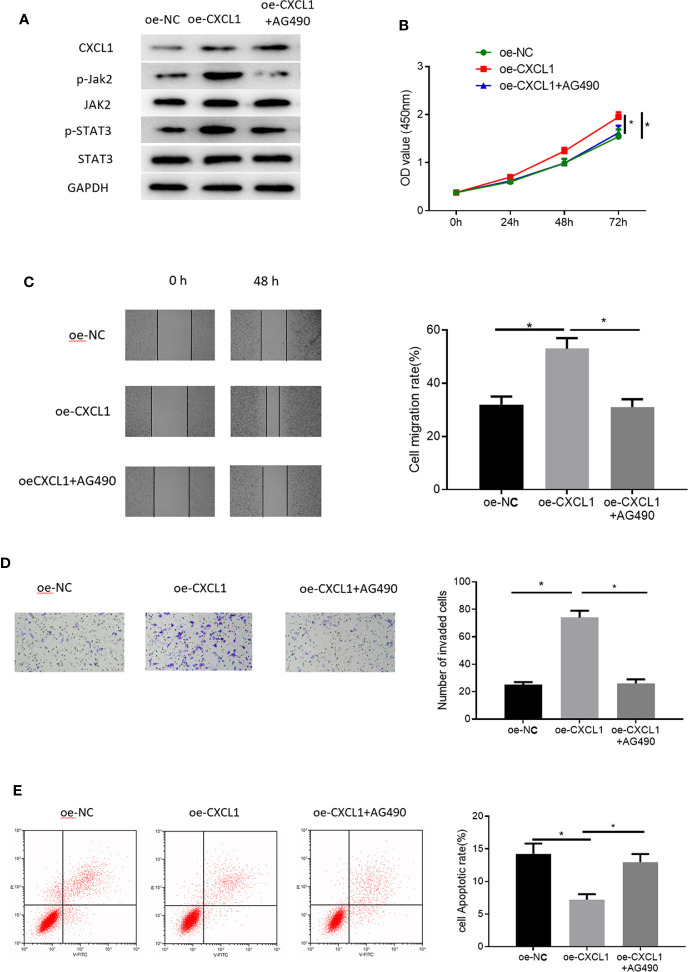
CXCL1 activates JAK-STAT signaling pathway to promote cell proliferation, migration, invasion and inhibit cell apoptosis of CRC. Cells were divided into oe-NC, oe-CXCL1, and oe-CXCL1+AG490 groups. **(A)** Western blot showed the protein levels of CXCL1 in SW480 cells and the levels of the key proteins involved in JAK-STAT signaling way (p-Jak1, Jak2, p-STAT3, and STAT3), **(B)** CCK-8, **(C)** wound healing assay, **(D)** Transwell invasion assay (100×), and **(E)** flow cytometry showed the proliferation, migration, invasion and apoptosis of SW480 cells in each group (*p < 0.05).

To gain more insight into the functional mechanism of CXCL1, KEGG database was consulted to identify related signaling pathways and it was found that CXCL1 as a cytokine could exert its regulatory function *via* JAK-STAT signaling pathway (**Supplementary Figure 3**). Many studies have reported that JAK-STAT signaling pathway is implicated in CRC proliferation and metastasis (19–21). Therefore, we reasoned that CXCL1 might modulate cell proliferation, migration, invasion and apoptosis of CRC *via* JAK-STAT signaling pathway, and we conducted some relevant experiments in succession for verification. Pathway inhibitor AG490 was used and the effect against JAK-STAT pathway was observed through Western blot experiments with the results plotted in **Supplementary Figure 4**. Thereafter, three groups were set for SW480 cells to clarify the regulatory mechanism by which CXCL1 regulates CRC cells *via* JAK-STAT signaling pathway: oe-NC, oe-CXCL1, and oe-CXCL1+AG490. Firstly, levels of key proteins involved in JAK-STAT signaling pathway including Jak2, STAT3, and their phosphorylated forms (p-Jak2 and p-STAT3) were detected, finding that p-Jak2 and p-STAT3 were significantly up-regulated in the oe-CXCL1 group relative to those in the oe-NC group. However, their levels were recovered after the oe-CXCL1 transfected cells were treated by AG490 (10 nM) for 24 h (oe-CXCL1+AG490 group) (**Figure 6A**). In view of this, it could be seen that CXCL1 was able to induce activation of JAK-STAT signaling pathway. Furthermore, the mechanism of CXCL1/JAK-STAT underlying cell biological behaviors of CRC was explored. As detected by CCK-8, wound healing assay, Transwell invasion assay and flow cytometry, overexpressing CXCL1 remarkably increased cell proliferation, migration and invasion but reduced cell apoptosis, which were attenuated when the cells were treated with AG490 (**Figures 6B–E**). These findings collectively elucidated that CXCL1 induced activation of JAK-STAT signaling pathway in turn promoting cell proliferation, migration, invasion, and inhibiting cell apoptosis.

### CXCL1 Promotes CRC Proliferation *In Vivo via* JAK-STAT Signaling Pathway

**Figure 7:**
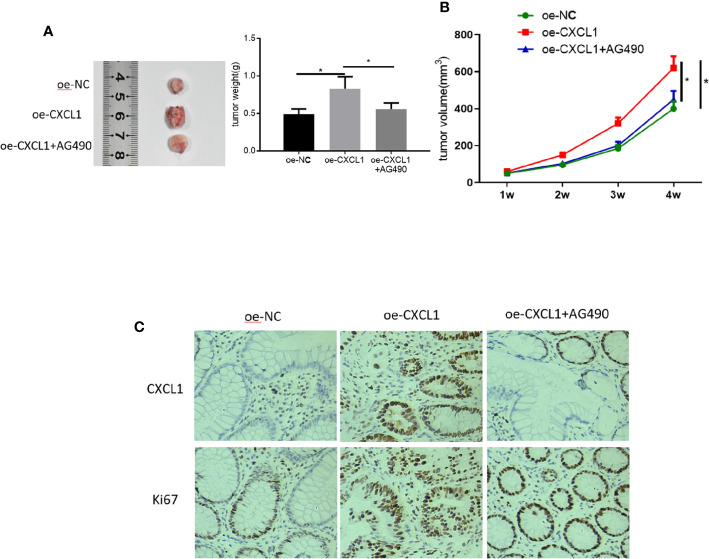
CXCL1 promotes CRC proliferation *in vivo via* JAK-STAT signaling pathway. SW480 cells transfected with oe-NC, oe-CXCL1 and oe-CXCL1+AG490 were subcutaneously grafted into nude mice, respectively. **(A)** Tumor size and tumor weight after 4 weeks, **(B)** tumor volume in the first 4 weeks, **(C)** immunohistochemistry showed the levels of CXCL1 and Ki67 in cells (*p < 0.05).

Tumor xenograft experiments were performed to clarify the role of CXCL1/JAK-STAT in CRC proliferation *in vivo*. SW480 cells transfected with oe-NC, oe-CXCL1, and oe-CXCL1+AG490 were subcutaneously grafted into nude mice, respectively. Tumor volume was calculated every week and the xenograft tumors were photographed and weighing after isolated (4 weeks later). Results plotted in **Figures 7A, B** showed that CXCL1 overexpression was beneficial for tumor growth *in vivo* but AG490 had the opposite effect. In addition, levels of CXCL1 and proliferation-related protein Ki67 were assessed using immunohistochemistry, revealing that both CXCL1 and Ki67 expression levels were much higher in the oe-CXCL1 group relative to those in the oe-NC group, whereas those in the oe-CXCL1+AG490 and the oe-NC groups were similar (**Figure 7C**). In sum, CXCL1 was proven to have the ability of potentiating CRC proliferation *in vivo via* JAK-STAT signaling pathway.

## Discussion

Chemokines, whose subunit molecular weight is about 8-15kDa, are a class of chemotactic cytokines and important members among all inflammatory mediators. The effects of chemokines on tumor are predominantly divided into two aspects: direct effect (regarding tumor malignant transformation, tumor survival and growth, invasion, and metastasis) and indirect effect (regarding angiogenesis, interaction between tumor and leukocytes) (22). In addition, according to the location of the conserved cysteine on amino terminal, chemokines can be classified into four families, including CXC, CC, CX3L, and C (X represents amino acid) (23). CXCL1 is a member of the CXC family and it is involved in multiple biological processes by specifically interacting with its receptor CXCR2, such as angiogenesis, inflammation, wound healing, tumorigenesis and development. It is reported that chemokines play vital roles in CRC. For instance, Li L et al. (24) suggested that chemokine CXCL7 could be used as a biomarker for CRC. Yu X et al. (25) discovered that chemokine CXCL12 or CXCR4 could activate RHoA signaling pathway to foster CRC progression. In addition, Gao YJ et al. (26) also revealed that down-regulation of chemokine CXCL11 is responsible for suppression of cell growth as well as epithelial-mesenchymal transition progress. In the present study, we applied bioinformatics analysis and found that CXCL1 was highly expressed in CRC. Meanwhile, consistent results were obtained when it comes to clinical CRC tissue samples and cells. Afterwards, we discussed the role of CXCL1 in CRC from the aspect of cell biological behaviors. It could be seen that CXCL1 was able to induce cell proliferation, migration, invasion, and inhibit cell apoptosis of CRC, which supports the necessity of CXCL1 study in CRC.

Increasing evidence has proven that miRNAs participate in cell proliferation, migration, invasion and metastasis of CRC. Zhu M et al. (27) discovered that miR-139-5p inhibitory functions on cell proliferation and invasion of CRC *via* decreasing nuclear factor-κB (NF-Kb) activity and in turn mediated chronic inflammation. Wang Y et al. (28) showed that lncRNA TTN-AS1 sponges miR-376a-3p to up-regulate KLF15 expression, consequently boosting CRC progression. Moreover, miR-215-3p is reported to play a negative role in cell growth, migration and invasion of CRC by interacting with its target FOXM1 (10). miR-302e is one of the 8 members of the miR-302 family able to suppress the proliferation of cervical squamous cell carcinoma and breast cancer, also to impede the angiogenesis and cell invasion of CRC (29–31). To make the functional mechanism of CXCL1 in CRC more specific, this study firstly used bioinformatics methods and identified that CXCL1 was a downstream target of miR-302e. For further verification, miR-302e was detected in CRC tissue and cells and it was found that there was a negative correlation between miR-302e and CXCL1 expression, which shows a good agreement with the established regulatory mechanism of miRNA-mRNA axis. Thereafter, miR-302e overexpression was established and it was found that level of CXCL1 was concomitantly reduced. In view of these, CXCL1 was negatively regulated by miR-302e. In addition, dual-luciferase reporter assay was conducted and further identified their direct targeting relationship. Finally, a series of experiments were performed to make a discussion on the role of miR-302e in CRC cell biological behaviors and rescue experiments were run for clarifying the underlying mechanism of the CXCL1/miR-302e axis. As expected, miR-302e inhibited cell proliferation, migration, and invasion but promoted cell apoptosis of CRC, and such effect could be attenuated by CXCL1 overexpression.

The Janus kinase signal transducer and activator of transcription (JAK-STAT) signaling pathway is a key player involved in the regulation between cytokines and growth factors, also implicates in cell development, differentiation, proliferation, and apoptosis (32). Other than the normal physiological functions, JAK-STAT signaling pathway also functions during tumorigenesis and development. For example, ELTD1 can activate the JAK-STAT3/HIF-1α axis to enhance cell proliferation, migration, and invasion of neuroglioma (33). miR-146a can induce the activation of JAK2-STAT3 pathway *via* down-regulating CNTFR, thereby affecting cell proliferation, migration, invasion and apoptosis in acute lymphatic leukemia and acute myeloid leukemia (34). Notably, JAK-STAT signaling pathway also functions in CRC. As reported, ITIH4-AS1 can be increased by decreased REST and serve as an oncogene in CRC *via* FUS in the case of JAK-STAT3 pathway activation (19). However, the relationship between CXCL1 and JAK-STAT signaling pathway has not been explored and our study would make sense. In the present study, we found that CXCL1 overexpression resulted in a significant elevation of p-Jak2 and p-STAT3 levels, and AG490 as an inhibitor of JAK-STAT signaling pathway reversed their levels. In addition, functional experiments including CCK-8, wound healing assay, Transwell invasion assay and flow cytometry were performed and also found that AG490 reversed the effect of CXCL1 overexpression on cell biological behaviors. Therefore, it could be seen that CXCL1 induced the activation of JAK-STAT signaling pathway in turn enhancing cell proliferation, migration, invasion and decreasing cell apoptosis of CRC. Furthermore, tumor xenograft experiments were conducted and indicated the positive effect of CXCL1 on CRC proliferation *in vivo via* JAK-STAT activation as well. In all, these findings fully demonstrate that CXCL1 implicates in the occurrence and development of CRC *via* the activation of JAK-STAT signaling pathway.

In conclusion, we validated that CXCL1 exerted its role in CRC by acting as an oncogene and it was directly mediated by miR-302e. Meanwhile, we found that CXCL1 could induce the activation of JAK-STAT signaling pathway and in turn promote cell proliferation, migration, invasion, and inhibit cell apoptosis. Overall, our study helps provide a specific molecular therapeutic target for CRC treatment, and our systemic description on the corresponding functional mechanisms makes it possible for treating metastasis of advanced CRC.

## Data Availability Statement

The original contributions presented in the study are included in the article/**Supplementary Material**. Further inquiries can be directed to the corresponding author.

## Ethics Statement

This study had gained the informed consent from each subject and been approved by the Ethic Committee of the Third Affiliated Hospital of Fujian University of Traditional Chinese Medicine. Participants gave their written informed consent.

## Author Contributions

All authors contributed to data analysis, drafting, and revising the article; gave final approval of the version to be published; and agreed to be accountable for all aspects of the work. All authors contributed to the article and approved the submitted version.

## Funding 

This study was supported by the Fujian Natural Science Foundation (program number: 2017J01322).

## Conflict of Interest

The authors declare that the research was conducted in the absence of any commercial or financial relationships that could be construed as a potential conflict of interest.

## Supplementary Material

Supplementary Figure 1 A heat map shows the 294 DEGs related to CRC identified in GEO database. The abscissa represents the samples and the ordinate represents the DEGs. Dendrogram (left) shows the clustering analysis of the DEGs.

Supplementary Figure 2 PPI analysis of the 294 CRC-related DEGs. Each node refers to a protein and the edge linking two nodes refers to protein-protein association. The node color refers to the degree (the darker the node color, the higher the degree).

Supplementary Figure 3 Chemokines and JAK signaling pathway.

Supplementary Figure 4 Western blot image for protein levels of CXCL1 and key proteins involved in JAK-STAT signaling pathway at the presence of pathway inhibitor AG490.
