# Supplementary figures and images for: CXCL1 Regulated by miR-302e Is Involved in Cell Viability and Motility of Colorectal Cancer via Inhibiting JAK-STAT Signaling Pathway
Source: Front Oncol. 2021 May 17;10:577229. doi: 10.3389/fonc.2020.577229 (PMC8166233; doi:10.3389/fonc.2020.577229)

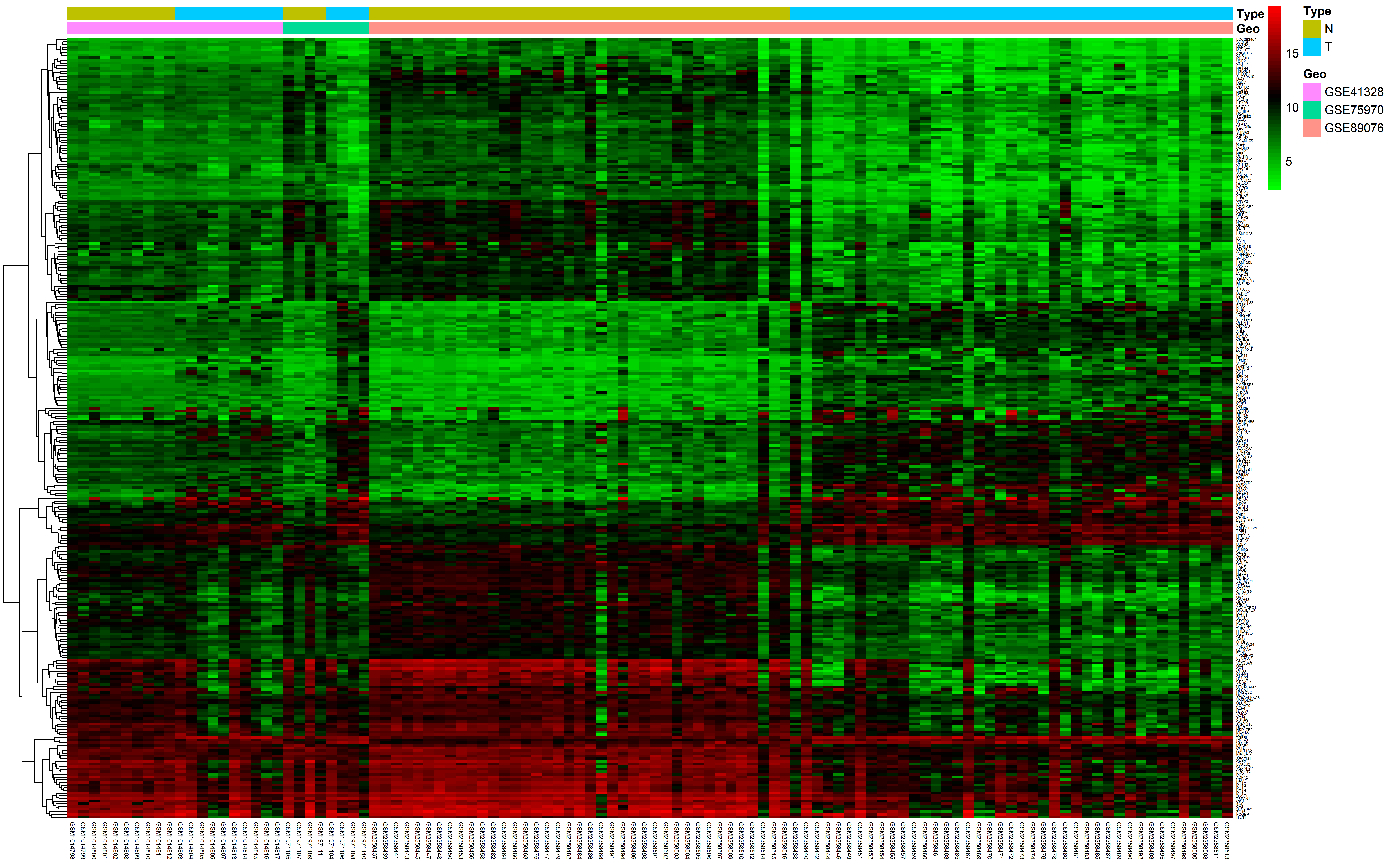

Supplement: Supplementary Figure 1 — A heat map shows the 294 DEGs related to CRC identified in GEO database. The abscissa represents the samples and the ordinate represents the DEGs. Dendrogram (left) shows the clustering analysis of the DEGs. [file Image_1.tiff]

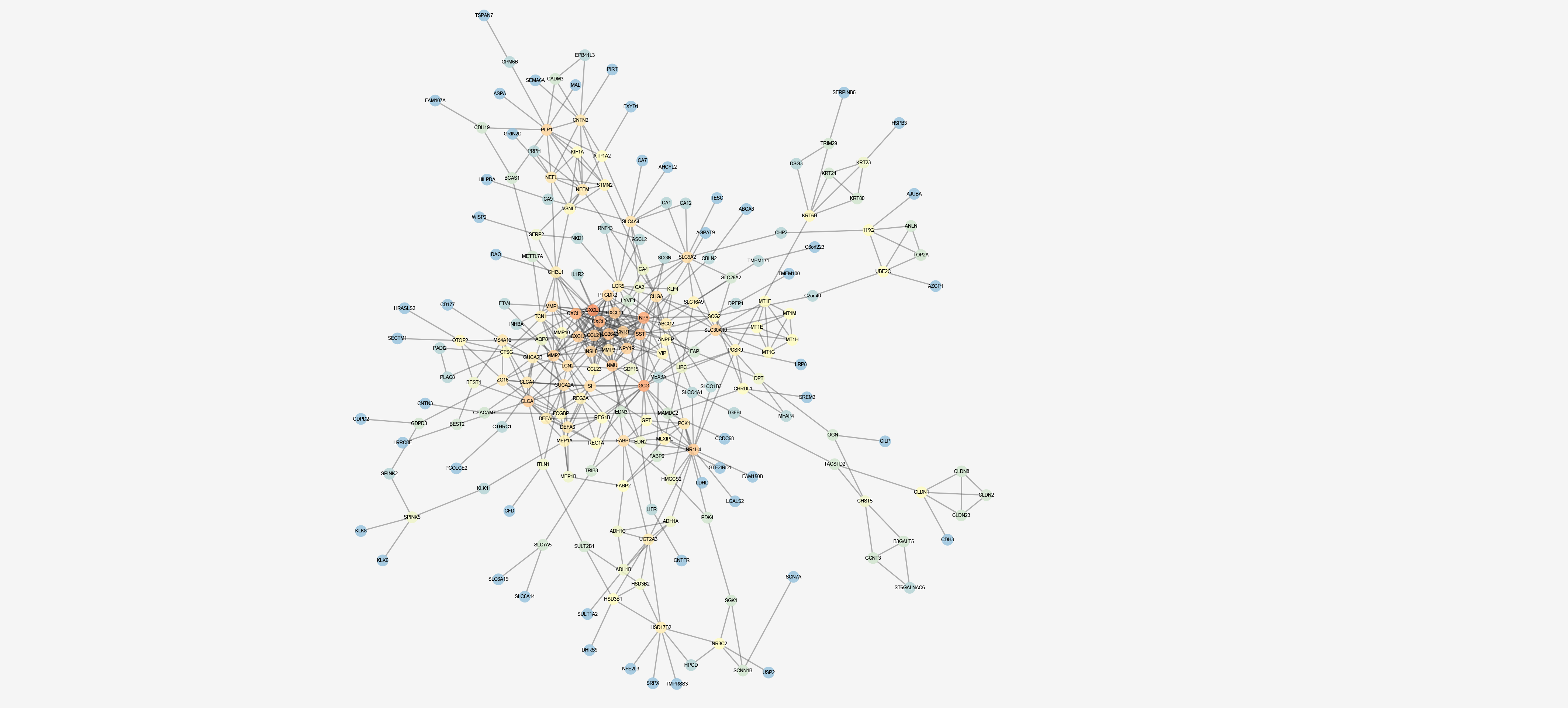

Supplement: Supplementary Figure 2 — PPI analysis of the 294 CRC-related DEGs. Each node refers to a protein and the edge linking two nodes refers to protein-protein association. The node color refers to the degree (the darker the node color, the higher the degree). [file Image_2.tif]

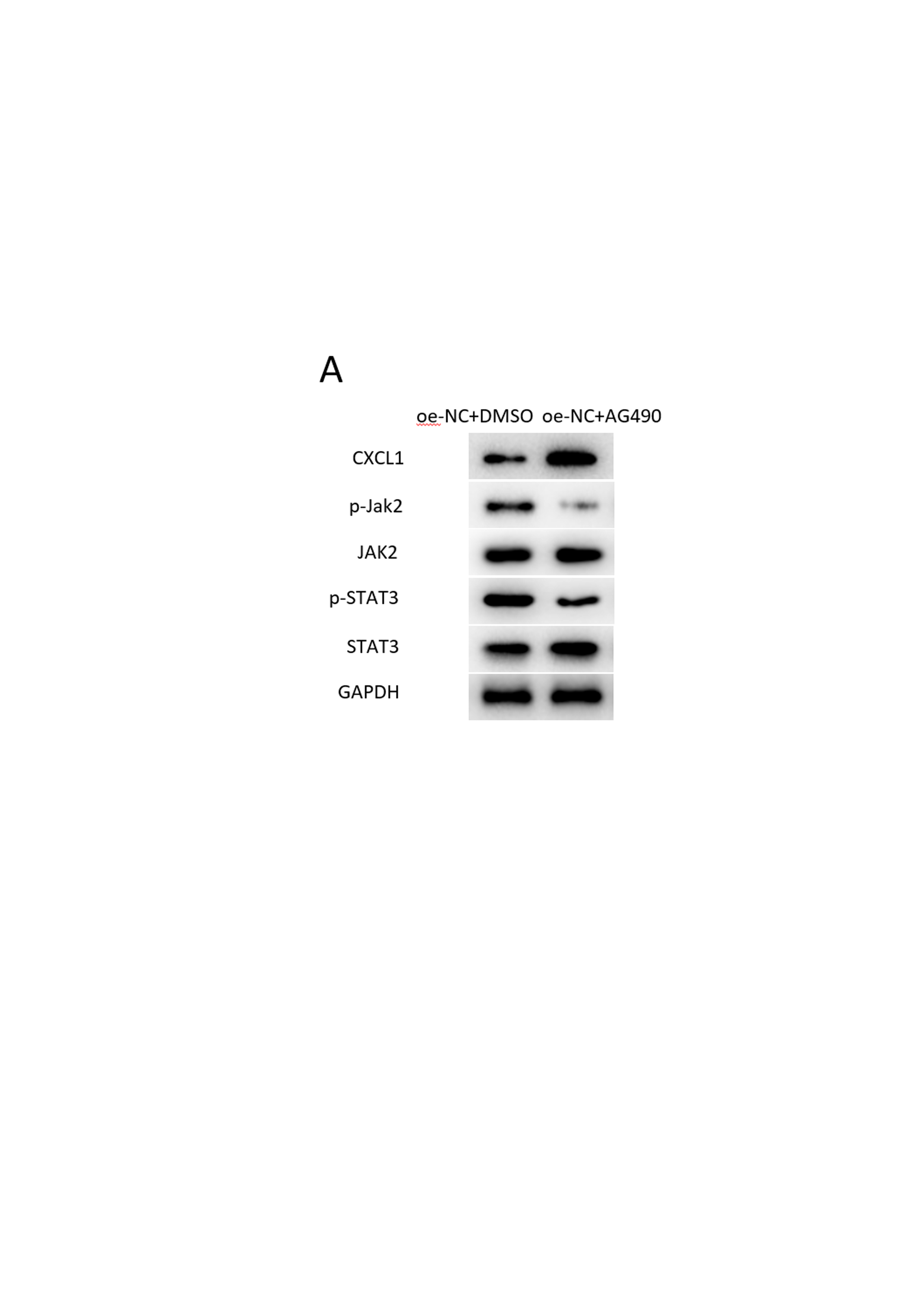

Supplement: Supplementary Figure 4 — Western blot image for protein levels of CXCL1 and key proteins involved in JAK-STAT signaling pathway at the presence of pathway inhibitor AG490. [file Image_4.tif]
